# Wider Potential Windows of Cellulose Multiwall Carbon Nanotube Fibers Leading to Qualitative Multifunctional Changes in an Organic Electrolyte

**DOI:** 10.3390/polym13244439

**Published:** 2021-12-17

**Authors:** Rudolf Kiefer, Fred Elhi, Anna-Liisa Peikolainen, Tarmo Tamm

**Affiliations:** 1 Conducting Polymers in Composites and Applications Research Group, Faculty of Applied Sciences, Ton Duc Thang University, Ho Chi Minh City 700000, Vietnam; 2 Intelligent Materials and Systems Lab, Institute of Technology, University of Tartu, Nooruse 1, 50411 Tartu, Estonia; elhi.fred@gmail.com (F.E.); anna.liisa.peikolainen@ut.ee (A.-L.P.); tarmo.tamm@ut.ee (T.T.)

**Keywords:** Cell-CNT fibers, linear actuation, organic electrolyte, change of actuation, potential range

## Abstract

The trend across the whole of society is to focus on natural and/or biodegradable materials such as cellulose (Cell) over synthetic polymers. Among other usage scenarios, Cell can be combined with electroactive components such as multiwall carbon nanotubes (CNT) to form composites, such as Cell-CNT fibers, for applications in actuators, sensors, and energy storage devices. In this work, we aim to show that by changing the potential window, qualitative multifunctionality of the composites can be invoked, in both electromechanical response as well as energy storage capability. Cell-CNT fibers were investigated in different potential ranges (0.8 V to −0.3 V, 0.55 V to −0.8 V, 1 V to −0.8 V, and 1.5 V to −0.8 V), revealing the transfer from cation-active to anion-active as the potential window shifted towards more positive potentials. Moreover, increasing the driving frequency also shifts the mode from cation- to anion-active. Scanning electron microscopy (SEM) and energy dispersive X-ray (EDX) spectroscopy were conducted to determine the ion species participating in charge compensation under different conditions.

## 1. Introduction

Smart materials are defined as materials with collaborative functionalities that either sense or respond to an external, typically electrical, signal [1]. The modern trend has been to create smart materials from natural, biodegradable, and biocompatible polymers, such as cellulose (Cell), to which electroactive materials such as carbon nanotubes (CNT) or other carbon materials such as graphene nanoplatelets [2] are added. Cellulose can be dissolved in ionic liquids [3], and the mixture with CNT can be processed into fibers through extrusion [4] or wet spinning [5]. The material is regenerated in an anti-solvent, for which water is in most cases applied [6]. Cell-CNT fiber composites can find applications in sensors [7], supercapacitors [8], other energy storage devices [9], smart textiles [10], and actuators [11,12]. The combination of hydroxypropyl cellulose with wax/halloysite hybrid microspheres formed nanocomposite films showing potential applications in heat and energy storage [13]. Numerous attempts have been made to apply CNT material or its cellulose composites in sensory applications, including for the detection of volatile organic compounds [14], water [15], or ion selectivity, mostly in aqueous solutions [16].

The basic electromechanical mechanism behind the actuation properties stems from the CNT inside the cellulose, whose electromechanical responsiveness was first introduced by Baughman et al. [17] and is brought about by the charging of CNT as in an electrochemical capacitor [18]. The charge injected into CNT is balanced by the electrical double layer, formed in a non-faradaic process of bringing ions from the surrounding electrolyte close to the CNT surface, where high charge concentration leads to changes in C–C covalent bonds [19]. Some researchers have also suggested a mixed non-faradaic and faradaic process [20] due to the observation of (apparent) oxidation and reduction waves in cyclic voltammetry. Some others have proposed that with more negative potential, the faradaic component is increased [21]. The CNT actuation mechanism has, therefore, still not been fully understood, and more basic research needs to be carried out.

To shed light on the processes and the overall electromechanical response of the composites as a function of driving potential and frequency, Cell-CNT fibers [22] are studied here in an organic electrolyte, to broaden the applicable potential window.

The stress and strain response of the fibers driven by cyclic voltammetry in potential ranges of 0.8 V to −0.3 V, 0.55 V to −0.8 V, 1.0 V to −0.8 V, and 1.5 V to −0.8 V, as well as square wave potential steps at frequencies from 2.5 mHz to 0.1 Hz, was determined. To investigate the sensor properties, chronopotentiometric measurements in potential ranges of 0.55 V to −0.8 V and 1.5 V to −0.8 V were performed, and the specific capacitances were determined. Flexible multifunctional materials such as Cell-CNT fiber can give a new direction to smart materials either as actuators or energy storage materials in wearable applications.

The Cell-CNT fibers were characterized using SEM micrographs of the cross-sections, and conductivity was determined before and after driving cycles. The ion content in positively and negatively charged states was determined by EDX spectroscopy.

## 2. Materials and Methods

### 2.1. Materials

Multiwall carbon nanotubes (MWCNT, Baytubes^®^ C150P; amorphous carbon content 0%) with an average outside diameter of 13 nm, inside diameter of 4 nm, and length over 1 μm were obtained from Bayer Material Science (Leverkusen, Germany) and used as supplied. 1-Ethyl-3-methylimidazolium chloride (EMIMCl, >97%), bis(trifluoromethane)sulfonimide lithium salt (LiTFSI, 99.9%), ethanol (technical), and propylene carbonate (PC, >99%) were purchased from Sigma-Aldrich (Taufkirchen, Germany). Deionized water Milli-Q+ (Tallinn, Estonia) was used as supplied.

### 2.2. Cell-CNT Fiber Formation

Cellulose was dissolved in EMIMCl, known for breaking down hydrogen bonds, and 50 wt.% MWCNT was dispersed in the solution by ultrasonication for 15 min (Ultrasonicator, Hielscher UP200S, Teltow, Germany). The suspension was filled in a syringe (0.76 mm inner diameter needle) and pressed into anti-solvent (Milli-Q+), forming cellulose multiwall carbon nanotube (Cell-CNT) fibers, as described in more detail recently [4]. The Cell-CNT fibers (at least three fibers from independent batches were studied) were washed several times with ethanol to remove excess of the ionic liquid EMIMCl and dried in an oven for 24 h at 40 °C (2 mbar). The diameter and length of the applied Cell-CNT fibers at different potential ranges was found to be in an average range of 0.98 mm × 0.44 mm, with the weight of fibers in the range of 1.2 ± 0.1 mg.

### 2.3. Electromechanical Deformation

The Cell-CNT fiber samples were cut into lengths of 4.4 ± 0.4 mm and stored for 24 h in the LiTFSI-PC electrolyte before measurements. The thickness of the Cell-CNT fibers was measured with a screw gauge (Eiscolab, Rochester, NY, USA) and found to be, in diameter, in the range of 0.98 ± 0.08 mm. The linear actuation of Cell-CNT fiber samples was performed on a linear analyzer setup with an in-house software [23], connected to a potentiostat (Biologic PG581, Seyssinet-Pariset, France), recording the mechanical changes as a response to the electrochemical signals in real time. The Cell-CNT fibers were fixed between a clamp with gold contacts (functioning as the working electrode) and a force sensor (TRI202PAD, Panlab, Barcelona, Spain) in a three-electrode cell with a platinum counter electrode and an Ag/AgCl (3 M KCl) reference electrode. The linear analyzer setup has two modes: one to measure the mass change (calculated to stress σ = weight x gravimetric constant g/fiber cylindrical area) at a constant fiber length of 1 mm (length between lower clamp and force sensor); the other to measure the length change of the fiber (calculated to strain ε = ΔL/L × 100%, ΔL = L − L_1_ with L being the original length and L_1_ the length change) with constant force of 0.5 mN applied. The linear muscle analyzer setup contains a movable stage to measure the stiffness k (mg μm^−1^) before the length change measurements, showing the stiffness of the Cell-CNT fiber to be in the range of 837 mg μm^−1^ (elastic modulus of 0.62 ± 0.05 MPa).

Before the measurement commenced, the Cell-CNT fibers were stretched in the linear muscle setup in a range of 0.1% for 12 h in 0.1 M LiTFSI-PC electrolyte. Cyclic voltammetry and square wave potential step measurements at 2.5 mHz to 0.1 Hz of fiber samples were performed applying one of the potential ranges: 0.8 V to −0.3 V, 0.55 V to −0.8 V, 1 V to −0.8 V, and 1.5 V to −0.8 V. Chronopotentiometric measurements were performed by applying current densities j and frequencies such as ±0.015 A g^−1^ (0.0025 Hz), ±0.03 A g^−1^ (0.005 Hz), ±0.06 A g^−1^ (0.01 Hz), ±0.15 A g^−1^ (0.025 Hz), ±0.3 A g^−1^ (0.05 Hz), and ±0.6 A g^−1^ (0.1 Hz). The charge density at each applied current density and frequency was constant at ±3 C g^−1^. From the potential time curves at each applied current density and frequency, the slopes of the discharging curves (after IR drop) were taken, and with Equation (1), the specific capacitance C_s_ was calculated [24].

(1) $$C_{s}=\frac{j}{-slope}$$

### 2.4. Characterization

Scanning electron microscopy (SEM) micrographs of the fiber surface and cross-section were recorded with a Vega Tescan (Tescan Orsay Holding, Brno-Kohoutovice, Czech Republic); energy dispersive X-ray spectroscopy (EDX, Oxford Instruments with X-Max 50 mm^2^ detector, High Wycombe, USA) was performed from the cross-sections after linear actuation measurements in charged and discharged state for each applied potential range after additional polarization at the final potential for 5 min. Raman spectroscopy applying a 514 nm argon-ion laser (Renishaw plc, resolution 2 cm^−1^, Wotton-under-Edge, UK) was performed for cellulose, Cell-CNT, and pristine CNT samples. The electronic conductivity (via resistivity R) of dried Cell-CNT fibers was determined directly after formation using a digital multimeter (LCR200 Meter, EXTECH instruments, Nashua, New Hampshire, USA). With the length l and the area A (cylindrical surface) of the fiber samples inserted in Equation (2), the electronic conductivity σ_e_ was calculated.

(2) $$\sigma_{e}=\frac{l}{R\;\cdot \;A}$$

## 3. Results and Discussion

As electromechanical transducers, Cell-CNT fibers can have potential applications in bending actuation [12] or linear actuation [22]. It has been observed recently that the electromechanical response of Cell-CNT fibers depends both quantitatively and qualitatively on the driving frequency as well as potential [22]. However, as in the present study the potential range was limited by the electrochemical window of water as the solvent, the full picture can be more complex. In organic solvents, the available electrochemical window is significantly larger; hence, it is expected that this will provide a much deeper understanding and perhaps allow us to establish a clearer understanding of the underlying mechanisms. The potential ranges 1–4 (0.8 V to −0.3 V, 0.55 V to −0.8 V, 1.0 V to −0.8 V, and 1.5 V to −0.8 V) were considered in this work. The choice of potential ranges covers limited negative charging (0.8 V to −0.3 V), limited positive charging (0.55 to −0.8 V), and increased positive charging at ranges 1.0 V to −0.8 V and 1.5 V to −0.8 V. EDX spectroscopy was performed from the cross-section of Cell-CNT fibers to establish the nature of the ions accompanying the positive and negative charging in each of the potential ranges. The characterization regarding FTIR was shown in previous research [22], and there was no difference whether 10 wt.% CNT was loaded or 50 wt.% as applied in this research.

### 3.1. Characterizations of Cell-CNT Fibers

**Figure 1:**
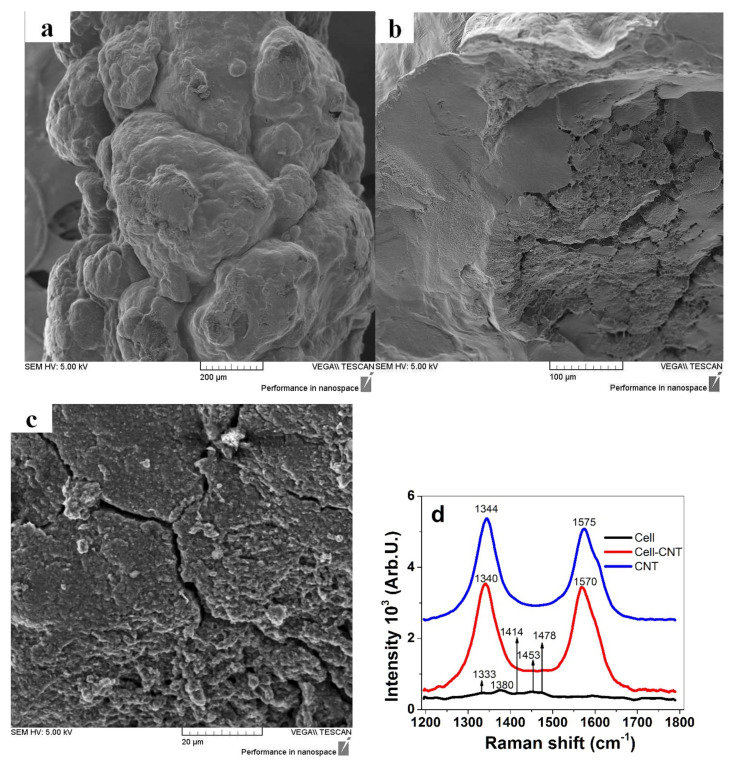
SEM micrographs (positively charged state), showing in (**a**) the Cell-CNT fiber surface (scale bar 200 μm) and in (**b**) the cross-section of the inner core (scale bar 100 μm), with magnification of the inner section (scale bar 20 μm) displayed in (**c**). Raman spectra (1800 cm^−1^–1200 cm^−1^, 514 nm, ion-argon laser) of cellulose (Cell, black line), Cell-CNT (red line), and pristine CNT are presented in (**d**).

The SEM micrograph of Cell-CNT fibers directly after formation is shown in Figure 1a. The cross-section of the inner core of the fiber in higher resolution before actuation measurements is presented in Figure 1b. Raman spectra of cellulose, Cell-CNT, and pristine CNT are shown in Figure 1c.

The fiber image of the CNT-Cell (Figure 1a) shows a bulky, rough-surfaced shape, similar to what has been observed before [25], owing to the fact that on the surface mostly cellulose is found. The cross-section of Cell-CNT fibers (Figure 1b) with higher magnification of the inner section shown in Figure 1c revealed no visible CNT clusters, but a compact section likely representing CNT surrounded by cellulose concentrated in the inner core; this is in contrast to the 10 wt.% MWCNT loaded fibers studied before [22]. The conductivity of all Cell-CNT samples was found to be in the range of 1.15 to 1.2 ± 0.1 mS cm^−1^, which is a nearly 6-fold improvement over the 10 wt.% CNT loading [22]. As a comparison, Cell-CNT with 4 wt.% CNT loading made by electro-spinning and with a much lower fiber diameter (in the range of 90 μm) showed a volume conductivity [14] of 0.8 mS cm^−1^.

**Figure 2:**
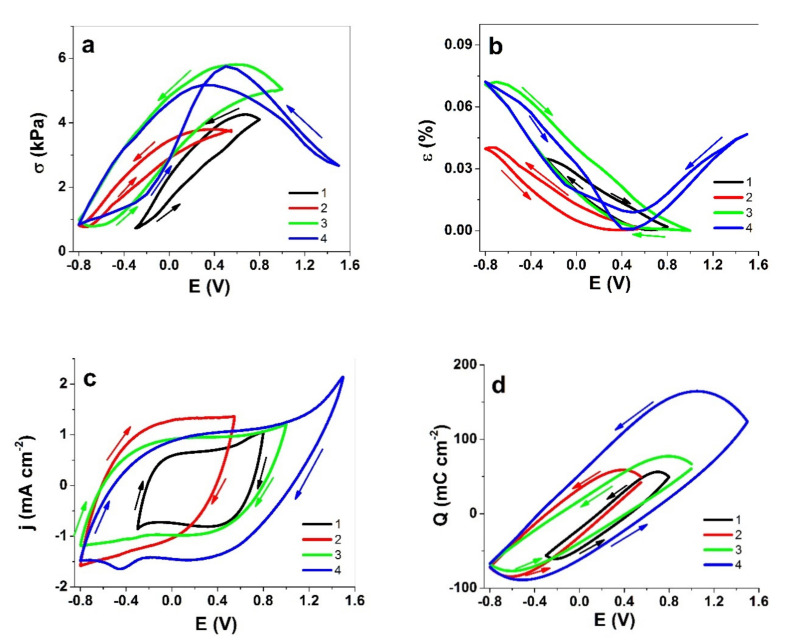
Cyclic voltammetry (scan rate 10 mV s^−1^) drives the linear actuation of Cell-CNT fibers in potential ranges 1 (0.8 V to −0.3 V, black curve), 2 (0.55 V to −0.8 V, red curve), 3 (1.0 V to −0.8 V, green curve), and 4 (1.5 V to −0.8 V, blue curve) in the LiTFSI-PC electrolyte. The results are presented in terms of (**a**) stress σ, (**b**) strain ε, (**c**) current density j, and (**d**) charge density Q against potential E. The arrows indicate the direction of the scans.

Raman shifts of cellulose shown in Figure 1d [26,27] belonging to C-H in plane bending vibrations are found at 1333 cm^−1^, and the 1380 cm^−1^ peak corresponds to the vibration deformation of the cellulose backbone, overlapped in Cell-CNT with the strong MWCNT peaks. The 1414 cm^−1^ peak (C–O–H), 1453 cm^−1^ peak (hydrogen bonding [28]), and the characteristic 1478 cm^−1^ peak (found in the literature at 1481 cm^−1^ [26]) belong to CH_2_ bending vibrations and are also observed in Cell-CNT with small shoulders. The pristine MWCNT (Figure 2a) with in-plane C–C bonds [29] are characterized by Raman shifts of the D peak at 1344 cm^−1^ and the G peak at 1575 cm^−1^. The peaks are shifted in Cell-CNT to a lower frequency with the D peak at 1340 cm^−1^ and G peak at 1570 cm^−1^, which was found to be the reason for CNT bundling at high loadings [30] shown with CNT centered in the middle of the fibers (Figure 1b). The ratios of intensities of I_D_/I_G_ peaks of pristine CNT and Cell-CNT, 1.06 and 1.03, respectively, were in a similar range, meaning no alterations to the CNT material had taken place during regeneration and formation into Cell-CNT fibers.

### 3.2. Electromechanical Response

The true mechanism of the electromechanical behavior of CNT composites has remained somewhat of a matter of dispute. There has been a lot of discussion in the literature about whether CNT material behaves as faradaic [20] or non-faradaic material. Other research on CNT yarns has pointed out that the size of the applied ions (either cations or anions) determines the direction as well the extent of stroke [31]. Not much has been reported regarding CNT composites’ actuation mechanism, with most attention given to non-faradaic processes [17]. In view of linear actuation, it is essential to have only one active species and actuation direction addressed, as mixed-mode actuation reduces the potential applications of such materials. Therefore, the electromechanical response as a function of driving potential range was studied. Two driving mechanisms, namely, cyclic voltammetry and square wave potential steps, in the frequency range of 2.5 mHz to 0.1 Hz were considered, to study the potential range effect also as a function of driving signal and frequency.

#### 3.2.1. Cyclic Voltammetry

**Figure 3:**
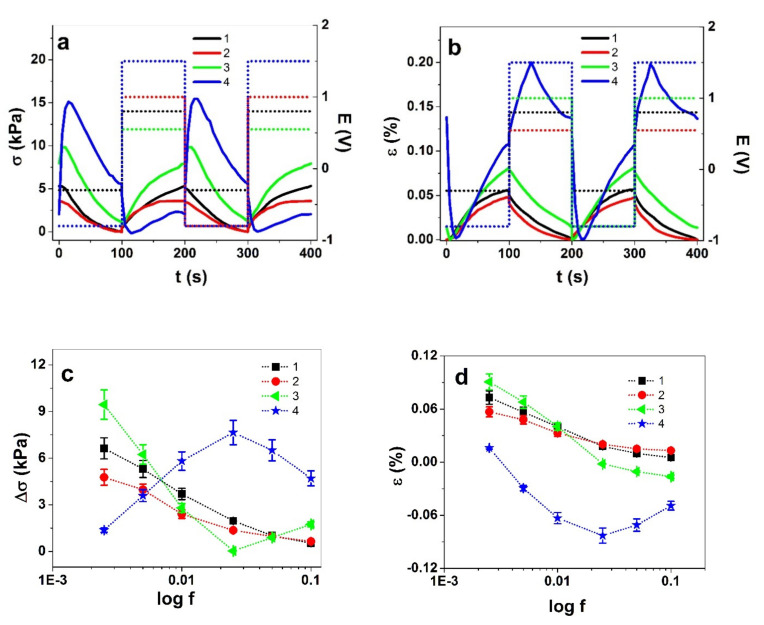
Square wave potential steps response of Cell-CNT fibers in the different potential ranges 1 (0.8 V to −0.3 V, ∙∙■∙∙, black line), 2 (0.55 V to −0.8 V, ∙∙●∙∙, red line), 3 (1.0 V to −0.8 V, ∙∙◄∙∙, green line), and 4 (1.5 V to −0.8 V, ∙∙★∙∙, blue line) in LiTFSI-PC electrolyte showing in (**a**) the stress σ and in (**b**) the strain ε against time at 5 mHz, two subsequent cycles (3–4). The stress difference Δσ and the strain ε at potential ranges 1–4 against frequency (log f) are presented in (**c**) and (**d**), respectively.

Cyclic voltammetric measurements of Cell-CNT fibers in different potential ranges were performed; the resulting stress and strain curves are presented in Figure 3a,b, respectively. The current density vs. potential curves are presented in Figure 3c, and the coulo-voltammetric results are shown in Figure 3d.

As seen from the stress and strain curves (Figure 2a,b), in potential ranges 1–3, there is major expansion at negative charging. The stress response is higher for potential ranges that reach higher positive values (0.8 V) but the clipping of negative potentials to −0.3 V appears to reduce the maximum strain response. While range 3 already hints at the introduction of mixed-mode response, with stress increasing both upon positive and negative polarization, it is clearly manifested in range 4, where strain response also has two maxima, one at either polarization. Mixed response modes reduce the (net) strain difference and stress difference values, and in general, are not beneficial for an electromechanical system. The cyclic voltammetry shapes in Figure 2c for potential ranges 1–3 correspond to typical capacitor-like behavior, while the potential range 4 shows a distinguishable wave at −0.46 V. As no coupled wave is found on the positive polarization side, it could be related to impurities left in the CNT materials [32].

With charge densities (Figure 2d), there was a clear relation between the charge density and the width of the potential window—the broader the window, the larger the charge exchanged, as expected. The largest increase was observed between ranges 3 and 4, explained by the 500 mV potential window width difference. Further studies with square wave potential steps were performed to investigate the responses of Cell-CNT fibers at different applied potential ranges and frequencies under a more abrupt driving regime.

#### 3.2.2. Square Wave Potential Steps

The square wave potential steps responses of Cell-CNT fibers as stress and strain at 5 mHz in different potential ranges are shown in Figure 3a,b. The current density curves at the same frequency are shown in Figure S1. The stress difference (absolute stress values) and strain-frequency dependence of Cell-CNT fibers are presented in Figure 3c,d. The charge densities in different potential ranges at each frequency upon positive and negative charging are presented in Figure S2a,b, respectively. For each potential range, at least three samples were measured; the results presented are the mean values. Positive strain represents expansion upon negative charging, while negative strain refers to expansion on positive charging.

As in the CV response above, the response to square wave potential steps (Figure 3a,b) in the potential ranges 1 and 2 shows the main expansion at negative charging with stress and strain slightly higher for potential range 1. The stress differences and strain in the potential ranges 1 and 2 decreased with increasing driving frequency, as can be expected.

In potential ranges 3 and 4, the more or less continuous trends in each cycle were replaced by the development of peaks (Figure 3a,d), which are significantly more dominant for potential range 4. Similar developments, alas to a much lesser extent, have also been observed before [22]. Carbide-derived carbon actuators in ionic liquids [33] have shown similar peaks, as well as to a lesser extent CNT fibers made by dielectrophoretic methods [34]. The switch from increasing to decreasing stress and strain during a single polarization step can only mean a shift in the composition of the fiber, or that of the double layer in particular.

**Figure 4:**
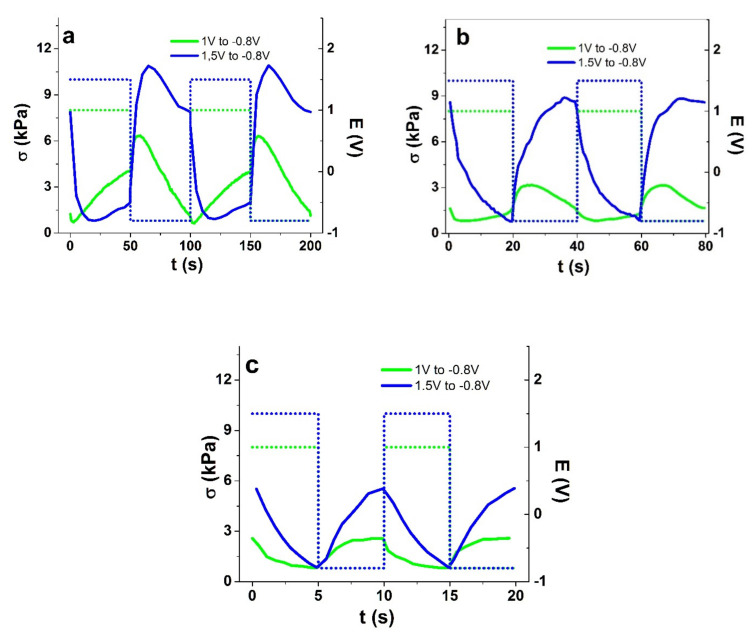
Square wave potential steps response of Cell-CNT fibers in potential range 3 (green curve) and potential range 4 (blue curve) showing stress σ against time in two subsequent cycles (3 to 4): (**a**) 10 mHz, (**b**) 25 mHz, and (**c**) 0.1 Hz.

In range 4, the main expansion is already at positive charging. Moreover, the change in response direction was not only observed during a potential step; it appeared also as a response to the change in driving frequency (Figure 3c,d). So, unlike the other potential ranges, in range 4 there was an increase in stress from 2.5 mHz to 25 mHz, while further up to 0.1 Hz the stress decreased. In range 3, the frequency dependence was non-monotonous as well, but with an initial decrease in stress difference until the same turning point at 25 mHz. While charge densities for potential range 3 were approximately 1.6 times higher compared with those in range 1, the potential range 4 showed 2-fold higher charge densities at low frequencies (Figure S2). To visualize the change in actuation direction in potential ranges 3 and 4, the respective stress response curves at 1 mHz, 25 mHz, and 0.1 Hz are shown in Figure 4a–c and the strain curves in Figure S3a–c.

In potential range 4 (Figure 4a–c), the stress response evolved with increasing driving frequency from mixed-mode with dominant expansion on positive charging at 0.01 Hz to virtually pure anion-dominated response by 0.1 Hz. In comparison, in potential range 3, the response at 0.01 Hz is dominated by cation-active expansion on negative charging, shifting to more and more expansion on positive charging with increasing frequency. Therefore, there is a clear shift towards the dominance of anions in the charge compensation with increasing driving frequency.

To elaborate on which mechanism is underlying such a change of response, the nature of the hydrophilic cellulose surrounding the hydrophobic CNT bundles in the core of the Cell-CNT fiber needs to be discussed. While it has been found that the aprotic PC solvent has only a slight influence on expansion rate [22], the behavior of the Li^+^ cations and TFSI^−^ anions needs to be considered here as well as the interaction of both ions during the positive/negative charging. The Li^+^ cations are solvated with 3–4 PC molecules [35], while the amphiphilic TFSI^−^ anions have been found to be very weakly solvated [36], assumed to be moving as a separate entity. Therefore, with increasing driving frequency, there is less and less time for a solvated cation to move in and out of position, and the role of charge compensation shifts to anions, which may have to rush in to compensate the charge of the remaining cations.

**Figure 5:**
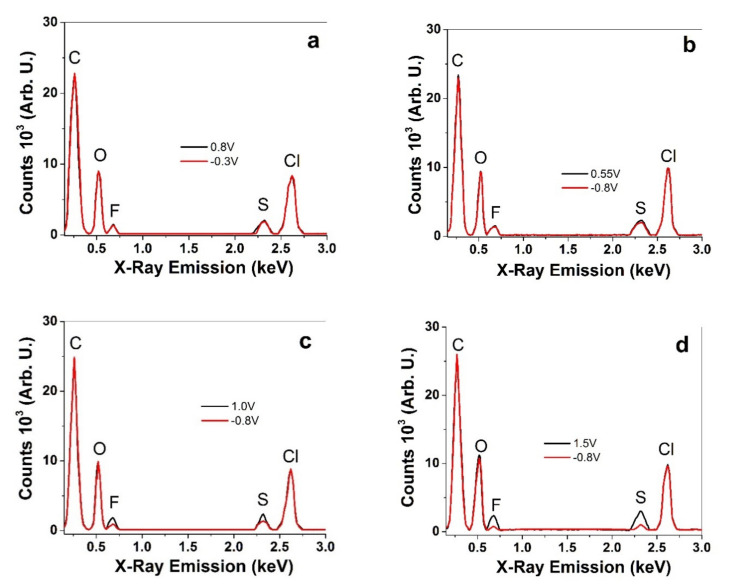
EDX spectra of cross-sections of Cell-CNT fibers after actuation cycles in positively charged state (5 min polarization, black line) and in negatively charged state (5 min, red line) showing in (**a**) potential range 1 (0.8 V to −0.3 V), (**b**) potential range 2 (0.55 V to −0.8 V), (**c**) potential range 3 (1.0 V to −0.8 V), and (**d**) potential range 4 (1.5 V to −0.8 V).

To gain more insight into which ions are participating during the charging/discharging process, EDX spectroscopy was performed for Cell-CNT fiber samples after linear actuation measurements. The EDX spectra were measured on the inner core of the Cell-CNT fiber (Figure 1c). The results are shown in Figure 5a–d.

All spectra in Figure 5 show a strong carbon peak (C) at 0.27 keV from CNT and cellulose and an oxygen peak (O) at 0.52 keV, which relate to the cellulose units. Peaks of lower intensities were found at 0.68 keV for fluorine (F) and at 2.32 keV for sulfur (S), which relate to the anion TFSI^−^ of the applied LiTFSI-PC electrolyte (Li^+^ is not detected by EDX). The chlorine (Cl) peak at 2.52 keV refers to the EMIMCl ionic liquid, which is still after several washing cycles partly present in the Cell-CNT fibers, as also seen previously [22]. The solvent applied here—PC—plays a role, as lower swelling than in the case of water [21] allows less residual EMIMCl to be removed. The EDX spectra of Cell-CNT after cycling in potential ranges 1 and 2 (Figure 5a,b) had no big change in charging/discharging lines with nearly the same intensities as fluorine and sulfur. In general, the existence of such peaks after negative polarization led to the conclusion that TFSI^−^ anions remain incorporated in the CNT bundles, and all charge compensation is due to Li^+^ flux. Higher charging, up to 1.0 V in the potential range 3, led to a decrease in fluorine and sulfur peaks after negative charging at −0.8 V. Further increase in charging potential to 1.5 V resulted in strong peaks of fluorine and sulfur, but a clear (nearly 70%) decrease in these peaks after negative polarization; hence, the anions are mobile enough to leave in these conditions. As the ion content in the material indicates the mobile species accompanying the charging and double layer formation, it also has a direct influence on the electromechanical response of the material.

In summary, it is not just the amplitude of stress or strain response of Cell-CNT fibers in the LiTFSI-PC electrolyte that depends on the potential range and the driving frequency, but also the “sign” of the response. The higher the potential range extends to the positive side, the larger is the part of the anions in the charge compensation, which is logical. Furthermore, the higher the driving frequency, the lower the potential required to switch from cation-dominated to anion-dominated charge compensation.

As any potential application in soft robotics or biomedical devices of Cell-CNT fibers likely would benefit from a single response direction, one needs to consider both the potential range and the driving frequency in order to ensure consistent and controllable response.

### 3.3. Energy Storage

CNT materials either in pristine fiber or as a composite with cellulose as shown here are known for their energy storage capability [37]. In order to determine the specific capacitance (Equation (1)), chronopotentiometric measurements were performed.

**Figure 6:**
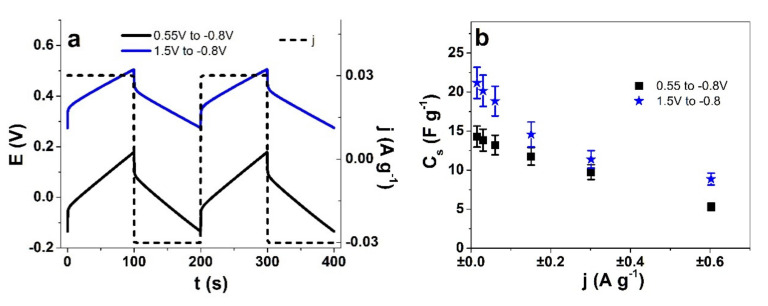
Chronopotentiometric measurements of Cell-CNT at current densities ± 0.015 A g^−1^ to ± 0.6 A g^−1^ (frequencies 2.5 mHz to 0.1 Hz) in LiTFSI-PC electrolyte. The potential time curves (2 subsequent cycles, 3–4) of Cell-CNT in the potential ranges 0.55 V to −0.8 V (black line) and 1.5 V to −0.8 V (blue line) are shown with current density j at ± 0.03 A g^−1^ (dashed line) at 5 mHz in (**a**). The specific capacitance C_s_ against applied current density j of Cell-CNT in potential ranges 0.55 V to −0.8 V (■) and 1.5 V to −0.8 V (★) is presented in (**b**).

As discussed above, the potential ranges 1 and 2 had main expansion upon negative charging with a virtually constant intensity of fluorine observed in EDX (Figure 5a,b); therefore, it must be assumed that the cations Li^+^ are the charge carriers. For potential range 4, the intensity of fluorine increases significantly upon positive charging; therefore, TFSI^−^ is mostly participating in the electrical double layer. The following analysis is shown for potential ranges 2 and 4, where in the linear actuation response analysis (Figure 3), expansion on negative charging (potential range 2, 0.55 V to −0.8 V) and expansion at positive charging (potential range 4, 1.5 V to −0.8 V) were observed, respectively. The potential time curves in the selected potential ranges 2 and 4 are shown in Figure 6a. The specific capacitance against applied current density is presented in Figure 6b.

The potential time curves of both the selected potential ranges (Figure 6a) revealed that there are differences in the shapes, with more positive voltage for range 4 (E_pos.charg_ 0.49 V, E_neg.charg._ 0.29 V) in comparison to range 2 (E_pos.charg._ 0.17 V, E_neg.charg._ −0.13 V). The discharging curves in Figure 6a of range 2 (0.55 V to −0.8 V) had a steeper slope, which influences the calculated specific capacitance (Equation (1)). The specific capacitance C_s_ for potential range 4 was at ±0.015 A g^−1^ in the range of 21 ± 2 F g^−1^, nearly 1.5 times higher than that of potential range 2 at 14 ± 1 F g^−1^. At each current density, the specific capacitance in the potential window 4 was higher. The values compare well with those of cellulose paper with aligned MWCNT [38] of 22 F g^−1^. In general, MWCNT-based materials [39] have shown specific capacitances in the range of 18–46 F g^−1^, while pristine MWCNT fiber made by dielectrophoresis [34] can reach 60 F g^−1^. CNT yarns have shown specific capacitance in the range of 23 F g^−1^ in an organic electrolyte [40]. Cellulose paper with aligned CNT [41] in 50 wt.% loadings has shown maximum specific capacitance in the range of 46 F g^−1^.

In summary, the Cell-CNT fibers can be considered truly multifunctional, showing electromechanical response varied by both potential range and driving frequency, but also as having energy storage capability with specific capacitance up to 21 F g^−1^.

Table 1 gives a comparison with previously studied electroactive Cell-CNT composite materials, in the form of fiber or other types of actuators.

Electrochemical actuators shown in Table 1 fall into categories of bending (in general a trilayer system) and linear length change. MWCNT can either form coatings or be inside the composites. High DC voltage is needed to achieve sufficient bending in air with either IL or ionic electrolyte membranes (polymer electrolyte). The actuation direction has been governed by anion influence with displacement at positive charging, with the exception of MWCNT fiber of dielectrophoretic formation (DEP) with cation-dominated actuation direction (expansion at negative charging). Several studies have seen mixed-mode response, and the differences have been explained by relative ion sizes [31]. While high absolute values in strain or specific capacitance were not aimed for, as the main focus was actuation direction control, the materials still compare favorably in several aspects. Further optimization can easily be performed, with increased conductivity as the main tool for higher performance.

## 4. Conclusions

The electromechanical response of Cell-CNT fibers in LiTFSI-PC as stress and strain was investigated as a function of driving potential range as well as driving regime and frequency. With potential ranges leaning on the negative potential side, the charge compensation was achieved largely by the flux of cations, as confirmed by the EDX analysis. As the driving potential range was extended towards positive potentials, increasing anion participation could be observed, being more pronounced if the driving regime is more abrupt in nature, like with the square potential steps as opposed to the more gradual cyclic voltammetry. Moreover, the shift to anion activity arrived sooner in potential ranges with increased driving frequency. Such perhaps unexpected behavior can be explained by the different solvation strengths of the anions and cations considered. Mixed-ion activity is typically best avoided in applications; therefore, depending on the driving frequency, some potential ranges are more favorable than others for consistent electromechanical response. The energy storage capacity of the Cell-CNT fibers was also found to be significant, adding to the beneficial multifunctional character of these remarkable composites.

## Figures and Tables

**Table 1:** Comparison of the electrochemical data of electroactive composites of cellulose with MWCNT and other combinations.

Electrochemical Actuators	Electrolyte Applied Voltage Current Density	Specific Capacitance (F g^−1^)	Actuation
Regenerated Cellulose + coating with MWCNT [12]	Ionic liquids 1.0 V to 0.0 V 0.25–0.4 mA cm^−2^ (20 mV s^−1^)	0.89 (20 mV s^−1^)	Trilayer, ±5 V Anion-dominated 0.7–1.5 cm displacement
Cellulose regenerated + MWCNT + rGO + MnO_2_ + PANI [42]	Ionic electrolyte membrane 1.0 V to 0.2 V 0.58 mA cm^−2^ (20 mV s^−1^)	1.4 (1 A g^−1^)	Trilayer, ±5 V 1.6 cm displacement Anion-dominated
MWCNT fiber Dielectrophoresis [34]	TBACF_3_SO_3_-PC 0.6 V to −0.55 V 1 mA cm^−2^,(5 mV s^−1^)	62 (0.2 A g^−1^)	Linear actuation Cation-dominated 0.1% strain
MWCNT yarn CVD, twisted [31]	TBAPF_6_/acetonitrile ±1.0 V 50 mV s^−1^	-	Linear actuation Mixed actuation −1.0 V: 0.035% strain +1.0 V: 0.065% strain
This work Regenerated Cellulose + 50 wt.% MWCNT fiber	LiTFSI-PC 1.5 V to −0.8 V (10 mV s^−1^) ~2 mA cm^−2^	21 (0.015 A g^−1^)	Linear actuation Mixed actuation −0.8 V: 0.07% +1.0 V: 0.05%

## Data Availability

The data presented in this study are available on request from the corresponding author.

## Supplementary Materials

The following are available online at https://www.mdpi.com/article/10.3390/polym13244439/s1, Figure S1: Current density time curves at frequency 0.005 Hz of Cell-CNT fiber in LiTFSI-PC electrolyte applied at different potential range 1 (0.8 V to −0.3V, black curve), potential range 2 (0.55 V to −0.8 V, red curve), potential range 3 (1.0 V to −0.8 V, green curve) and potential range 4 (1.5 V to −0.8 V, blue curve), Figure S2: Square wave steps of Cell-CNT fibers in LiTFSI-PC electrolyte at different potential ranges 1–4 with 1 (0.8 V to −0.3 V, ∙∙■∙∙), 2 (0.55 V to −0.8 V, ∙∙●∙∙), 3 (1.0 V to −0.8 V, ∙∙◄∙∙) and 4 (1.5 V to −0.8V, ∙∙★∙∙) showing in a: charge densities at positive charging Q_charg_ and in b: charge densities at negative charging Q_discharg_ against a logarithmic scale of frequencies (0.0025 Hz−0.1 Hz), Figure S3: Square wave steps of Cell-CNT fibers in a potential range 3 (green curve) and potential range 4 (blue curve) showing strain ε of two subsequent cycles (3rd to 4th) against time t at different frequencies in a: 10 mHz, b: 25 mHz and c: 0.1 Hz.

## References

[1] Jang Y., Kim S.M., Spinks G.M., Kim S.J. (2019). Carbon Nanotube Yarn for Fiber-Shaped Electrical Sensors, Actuators, and Energy Storage for Smart Systems. Adv. Mater..

[2] Amri M.R., Yasin F., Abdullah L.C., Al-Edrus S.S.O., Mohamad S.F. (2021). Ternary Nanocomposite System Composing of Graphene Nanoplatelet, Cellulose Nanofiber and Jatropha Oil Based. Polymers.

[3] Pinkert A., Marsh K.N., Pang S., Staiger M.P. (2009). Ionic liquids and their interaction with cellulose. Chem. Rev..

[4] Elhi F., Aid T., Koel M. (2016). Ionic liquids as solvents for making composite materials from cellulose. Proc. Est. Acad. Sci..

[5] Mo M., Chen C., Gao H., Chen M., Li D. (2018). Wet-spinning assembly of cellulose nanofibers reinforced graphene/polypyrrole microfibers for high performance fiber-shaped supercapacitors. Electrochim. Acta.

[6] Gupta K.M., Hu Z., Jiang J. (2013). Molecular insight into cellulose regeneration from a cellulose/ionic liquid mixture: Effects of water concentration and temperature. RSC Adv..

[7] Schroeder V., Savagatrup S., He M., Lin S., Swager T.M. (2019). Carbon nanotube chemical sensors. Chem. Rev..

[8] Jyothibasu J.P., Wang R.H., Ong K., Ong J.H.L., Lee R.H. (2021). Cellulose/carbon nanotube/MnO2 composite electrodes with high mass loadings for symmetric supercapacitors. Cellulose.

[9] Gui Z., Zhu H., Gillette E., Han X., Rubloff G.W., Hu L., Lee S.B. (2013). Natural cellulose fiber as substrate for supercapacitor. ACS Nano.

[10] Jing C., Liu W., Hao H., Wang H., Meng F., Lau D. (2020). Regenerated and rotation-induced cellulose-wrapped oriented CNT fibers for wearable multifunctional sensors. Nanoscale.

[11] Kim J., Kang Y., Ounaies Z., Bae S.H., Yun S. (2005). Electroactive paper materials coated with carbon nanotubes and conducting polymers. Am. Soc. Mech. Eng. Aerosp. Div. AD.

[12] Sun Z., Yang L., Zhang D., Song W. (2019). High performance, flexible and renewable nano-biocomposite artificial muscle based on mesoporous cellulose/ionic liquid electrolyte membrane. Sens. Actuators B Chem..

[13] Lisuzzo L., Caruso M.R., Cavallaro G., Milioto S., Lazzara G. (2021). Hydroxypropyl Cellulose Films Filled with Halloysite Nanotubes/Wax Hybrid Microspheres. Ind. Eng. Chem. Res..

[14] Qi H., Schulz B., Vad T., Liu J., Mäder E., Seide G., Gries T. (2015). Novel Carbon Nanotube/Cellulose Composite Fibers as Multifunctional Materials. ACS Appl. Mater. Interfaces.

[15] Qi H., Mäder E., Liu J. (2013). Unique water sensors based on carbon nanotube-cellulose composites. Sens. Actuators B Chem..

[16] Cao D., Pang P., Liu H., He J., Lindsay M. (2012). Electronic sensitivity of a single-walled carbon nanotube to internal electrolyte composition. Nanotechnology.

[17] Baughman R.H., Cui C., Zakhidov A.A., Iqbal Z., Barisci J.N., Spinks G.M., Wallace G.G., Mazzoldi A., De Rossi D., Rinzler A.G. (1999). Carbon nanotube actuators. Science.

[18] Kosidlo U., Omastova M., Micusik M., Ciric-Marjanovic G., Randriamahazaka H., Wallmersperger T., Aabloo A., Kolaric I., Bauernhansl T. (2013). Nanocarbon based ionic actuators-a review. Smart Mater. Struct..

[19] Pietronero L., Strässler S. (1981). Bond-Length Change as a Tool to Determine Charge Transfer and Electron-Phonon Coupling in Graphite Intercalation Compounds. Phys. Rev. Lett..

[20] Otero T.F., Martinez J.G., Asaka K. (2016). Faradaic and capacitive components of the CNT electrochemical responses. Front. Mater..

[21] Riemenschneider J., Temmen H., Monner H.P. (2007). CNT based actuators: Experimental and theoretical investigation of the in-plain strain generation. J. Nanosci. Nanotechnol..

[22] Elhi F., Peikolainen A.L., Kiefer R., Tamm T. (2020). Cellulose-multiwall carbon nanotube fiber actuator behavior in aqueous and organic electrolyte. Materials.

[23] Harjo M., Tamm T., Anbarjafari G., Kiefer R. (2019). Hardware and Software Development for Isotonic Strain and Isometric Stress Measurements of Linear Ionic Actuators. Polymers.

[24] Kaempgen M., Chan C.K., Ma J., Cui Y., Gruner G. (2009). Printable thin film supercapacitors using single-walled carbon nanotubes. Nano Lett..

[25] Yang L., Sun Z., Li F., Du S., Song W. (2019). Performance enhancement of cellulose based biocomposite ionic actuator by doping with MWCNT. Appl. Phys. A.

[26] Zhang K., Feldner A., Fischer S. (2011). FT Raman spectroscopic investigation of cellulose acetate. Cellulose.

[27] Agarwal U.P., Atalla R.H., Conners T.E., Banerjee S. (1995). Raman Spectroscopy. Surface Analysis of Paper.

[28] Lucas M., Wagner G.L., Nishiyama Y., Hanson L., Samayam I.P., Schall C.A., Langan P., Rector K.D. (2011). Reversible swelling of the cell wall of poplar biomass by ionic liquid at room temperature. Bioresour. Technol..

[29] Baskaran D., Mays J.W., Bratcher M.S., Uni V., Hall B., Knox V., December R.V., Re V., Recei M., March V. (2005). Noncovalent and Nonspecific Molecular Interactions of Polymers with Multiwalled Carbon Nanotubes. Chem. Mater..

[30] Liu Y., Kumar S. (2014). Polymer/carbon nanotube nano composite fibers-A review. ACS Appl. Mater. Interfaces.

[31] Foroughi J., Spinks G. (2019). Carbon nanotube and graphene fiber artificial muscles. Nanoscale Adv..

[32] Lyon J.L., Stevenson K.J. (2007). Anomalous electrochemical dissolution and passivation of iron growth catalysts in carbon nanotubes. Langmuir.

[33] Kaasik F., Tamm T., Hantel M.M., Perre E., Aabloo A., Lust E., Bazant M.Z., Presser V. (2013). Anisometric charge dependent swelling of porous carbon in an ionic liquid. Electrochem. Commun..

[34] Plaado M., Kaasik F., Valner R., Lust E., Saar R., Saal K., Peikolainen A., Aabloo A., Kiefer R. (2015). Electrochemical actuation of multiwall carbon nanotube fiber with embedded carbide-derived carbon particles. Carbon.

[35] Ikezawa Y., Ariga T. (2007). In situ FTIR spectra at the Cu electrode/propylene carbonate solution interface. Electrochim. Acta.

[36] Chaban V. (2015). Solvation of the fluorine containing anions and their lithium salts in propylene carbonate and dimethoxyethane. J. Mol. Model..

[37] Zhang L., Zhao X.S. (2009). Carbon-based materials as supercapacitor electrodes. Chem. Soc. Rev..

[38] Pushparaj V.L., Manikoth S.M., Ashavani K., Saravanababu M., Lijie C., Robert V., Linhardt R.J., Nalamasu O., Ajayan P.M. (2007). Flexible energy storage devices based on nanocomposite paper. Proc. Natl. Acad. Sci. USA.

[39] Felhősi I., Keresztes Z., Marek T., Pajkossy T. (2020). Properties of electrochemical double-layer capacitors with carbon-nanotubes-on-carbon-fiber-felt electrodes. Electrochim. Acta.

[40] Mirfakhrai T., Oh J., Kozlov M., Fok E.C.W., Zhang M., Fang S., Baughman R.H., Madden J.D.W. (2007). Electrochemical actuation of carbon nanotube yarns. Smart Mater. Struct..

[41] Pang Z., Sun X., Wu X., Nie Y., Liu Z., Yue L. (2015). Fabrication and application of carbon nanotubes/cellulose composite paper. Vacuum.

[42] Sun Z., Yang L., Zhao J., Song W. (2020). Natural Cellulose-Full-Hydrogels Bioinspired Electroactive Artificial Muscles: Highly Conductive Ionic Transportation Channels and Ultrafast Electromechanical Response. J. Electrochem. Soc..

