# Supplementary material for: Wider Potential Windows of Cellulose Multiwall Carbon Nanotube Fibers Leading to Qualitative Multifunctional Changes in an Organic Electrolyte
Source: Polymers (Basel). 2021 Dec 17;13(24):4439. doi: 10.3390/polym13244439 (PMC8708784; doi:10.3390/polym13244439)
Supplement: Supplementary file 1 [file polymers-13-04439-s001.zip › polymers-1473347-supplementary.pdf]

polymers

MDPI

Supplementary

Wider Potential Windows of Cellulose Multiwall Carbon Nanotube Fibers Leading to Qualitative Multifunctional Changes in an Organic Electrolyte

Rudolf Kiefer [1,*], Fred Elhi [2], Anna-Liisa Peikolainen [2] and Tarmo Tamm [2]

[1] Conducting Polymers in Composites and Applications Research Group, Faculty of Applied Sciences, Ton Duc Thang University, Ho Chi Minh City 700000, Vietnam
[2] Intelligent Materials and Systems Lab, Institute of Technology, University of Tartu, Nooruse 1, 50411 Tartu, Estonia; elhi.fred@gmail.com (F.E.); anna.liisa.peikolainen@ut.ee (A.-L.P.); tarmo.tamm@ut.ee (T.T.)
* Corresponding: rudolf.kiefer@tdtu.edu.vn; Tel: +886-905-60-55-15

Citation: Kiefer, R.; Elhi, F.; Peikolainen, A.-L.; Tamm, T. Wider Potential Windows of Cellulose Multiwall Carbon Nanotube Fibers Leading to Qualitative Multifunctional Changes in an Organic Electrolyte. *Polymers* **2021**, *13*, x. https://doi.org/10.3390/xxxxx

Academic Editor(s): Francisco Javier Espinach Orús and Quim Tarrés Farrés

Received: 5 November 2021
Accepted: 13 December 2021
Published:

Publisher's Note: MDPI stays neutral with regard to jurisdictional claims in published maps and institutional affiliations.

Copyright: © 2021 by the authors. Submitted for possible open access publication under the terms and conditions of the Creative Commons Attribution (CC BY) license (https://creativecommons.org/licenses/by/4.0/).

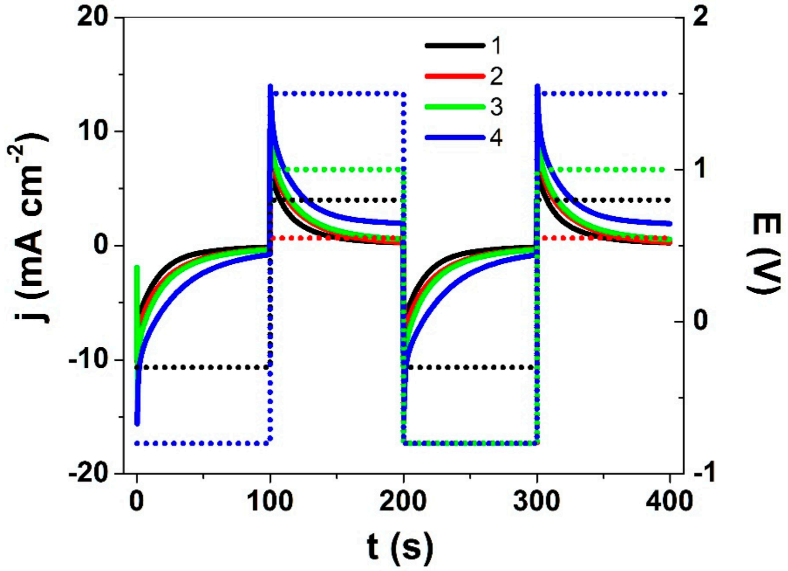

Figure S1. Current density time curves at frequency 0.005Hz of Cell-CNT fiber in LiTFSI-PC electrolyte applied at different potential range 1 (0.8V to -0.3V, black curve), potential range 2 (0.55V to -0.8V, red curve), potential range 3 (1.0V to -0.8V, green curve) and potential range 4 (1.5V to -0.8V, blue curve).

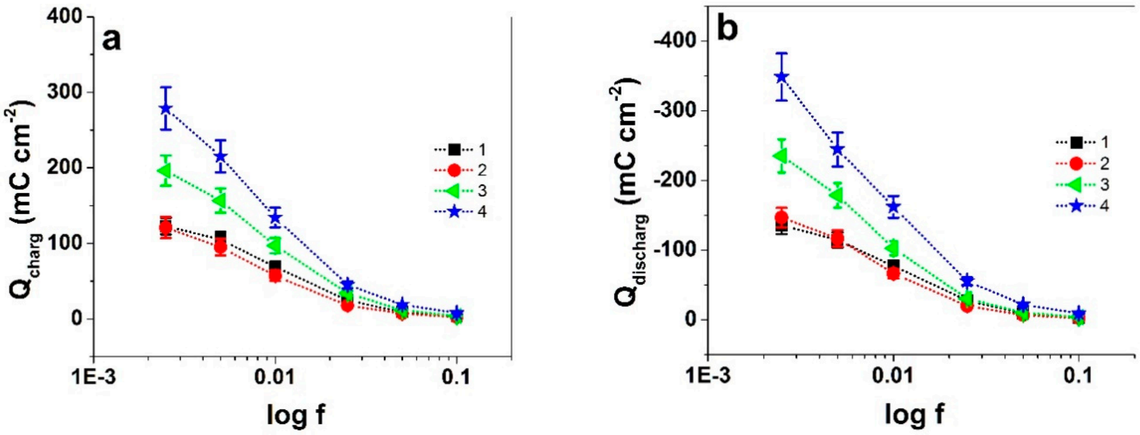

Figure S2. Square wave steps of Cell-CNT fibres in LiTFSI-PC electrolyte at different potential ranges 1-4 with 1 (0.8V to -0.3V, ··■··), 2 (0.55V to -0.8V, ··●··), 3 (1.0V to -0.8V, ··◀··) and 4 (1.5V to -0.8V, ··★··) showing in a: charge densities at positive charging Q_{charg} and in b: charge densities at negative charging $Q_{discharg}$ against a logarithmic scale of frequencies (0.0025Hz -0.1Hz).

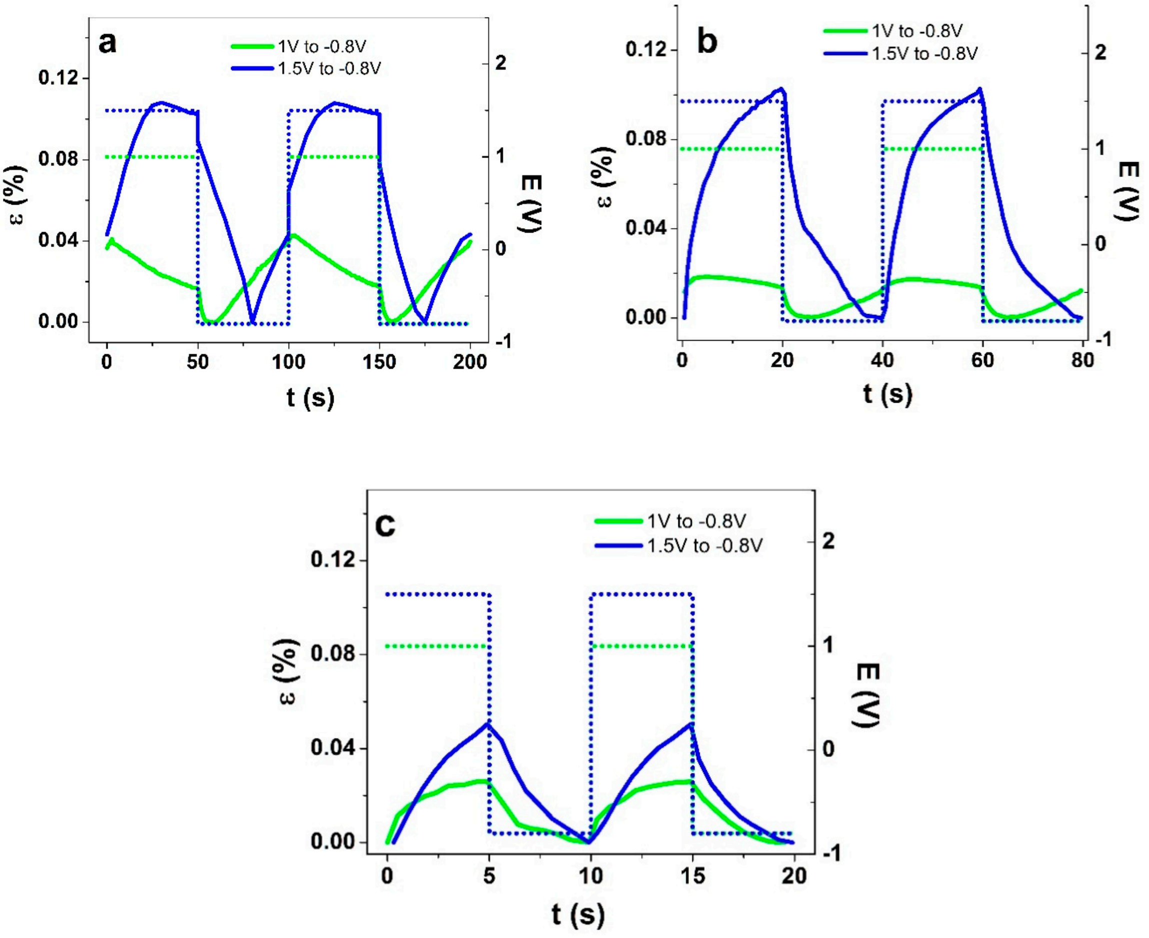

Figure S3. Square wave steps of Cell-CNT fibers in a potential range 3 (green curve) and potential range 4 (blue curve) showing strain ε of two subsequent cycles (3rd to 4th) against time t at different frequencies in a: 10mHz, b: 25mHz and c: 0.1Hz.
